# Concomitant mitral valve surgery with aortic valve replacement: a 21-year experience with a single mechanical prosthesis

**DOI:** 10.1186/1749-8090-2-24

**Published:** 2007-05-24

**Authors:** Niall C McGonigle, J Mark Jones, Pushpinder Sidhu, Simon W MacGowan

**Affiliations:** 1 Department of Cardiac Surgery, Royal Victoria Hospital, Grosvenor Road, Belfast, UK

## Abstract

**Background:**

Long-term survival for combined aortic and mitral valve replacement appears to be determined by the mitral valve prosthesis from our previous studies. This 21-year retrospective study assess long-term outcome and durability of aortic valve replacement (AVR) with either concomitant mitral valve replacement (MVR) or mitral valve repair (MVrep). We consider only a single mechanical prosthesis.

**Methods:**

Three hundred and sixteen patients underwent double valve replacement (DVR) (n = 273) or AVR+MVrep (n = 43), in the period 1977 to 1997. Follow up of 100% was achieved via telephone questionnaire and review of patients' medical records. Actuarial analysis of long-term survival was determined by Kaplan-Meier method. The Cox regression model was used to evaluate potential predictors of mortality.

**Results:**

There were seventeen cases (5.4%) of early mortality and ninety-six cases of late mortality. Fifteen-year survival was similar in both groups at 44% and 57% for DVR and AVR+MVrep respectively. There were no significant differences in valve related deaths, anticoagulation related complications, or prosthetic valve endocarditis between the groups. There were 6 cases of periprosthetic leak in the DVR group. Sex, pre-operative mitral and aortic valve pathology or previous cardiac surgery did not significantly affect outcome.

**Conclusion:**

The mitral valve appears to be the determinant of survival following double valve surgery and survival is not significantly influenced by mitral valve repair.

## Background

Replacement of the aortic valve at the time of double valve surgery is not contentious. However, the relative merits of either mitral valve repair or mitral valve replacement in these patients remains to be elucidated. In our previous study over 13 years with the St. Jude Medical (SJM) mechanical prosthesis valve, we observed that long-term survival for combined aortic and mitral valve replacement was similar to that for mitral valve replacement alone [1]. Therefore, we decided to include in present study all patients with a SJM aortic valve replacement (AVR), and concomitant mitral valve repair (MVrep) or mitral valve replacement (MVR) in a 21-year period to determine long-term survival for combined procedures. This study considered only patients in whom the SJM prosthesis was used for valve replacement.

## Methods

Three hundred and sixteen patients (227 female; 89 male) had AVR with either mitral valve replacement (DVR n = 273) or mitral valve repair (AVR+MVrep n = 43), between January 1977 and December 1997. All prosthetic valves were St. Jude Medical (SJM) mechanical prosthesis (St. Jude Medical, St. Paul. Mn.)

In the MVrep group, 28 patients had open mitral commisurotomy and 15 patients annuloplasty representing mitral stenosis and rheumatic fever as the major cause of mitral valve disease in this group.

The New York Heart Association (NYHA) functional classification III or IV was similar in each group (DVR 55.7%; AVR +MVrep 67.4%) (p = NS). The median age of patients undergoing DVR was 60.1 + 9.4 and 57.9 + 8.6 in the AVR + MVrep group (p = NS).

Patient clinical characteristics and pre-operative valve pathophysiology are shown in Table 1.

**Table 1:** Patient Characteristics

	**DVR**	**AVR+Mvrep**	**p**
**n**	273	43	
**Age **(median ± SD)	60.1 ± 9.4	57.9 ± 8.6	0.13
**Female**	197(72.2%)	30(70%)	0.72
**NYHA III+IV**	152(55.7%)	29(67.4%)	0.18
			
**Valvular Pathology**			
Rheumatic	211(77.3%)	31(72.1%)	0.44
Degenerative	56(20.5%)	8(18.6%)	>0.999
Ischaemic	4(1.5%)	1(2.3%)	0.52
Endocarditis	2(0.7%)	3(7.0%)	0.02
			
**Mitral valve lesion**			
Stenosis	94(34.4%)	21(48.8%)	0.088
Regurgitation	73(26.7%)	9(20.9%)	0.46
Mixed	99(36.3%)	7(16.3%)	0.009
Unknown	7(2.6%)	6(14%)	
			
**Aortic valve lesion**			
Stenosis	66(24.2%)	16(37.2%)	0.09
Regurgitation	88(32.2%)	9(20.9%)	0.16
Mixed	113(41.4%)	16(37.2%)	0.74
Unknown	6(2.2%)	2(4.7%)	
			
**Patient Years**	1993	381	

NYHA = New York Heart Association

Rheumatic or degenerative pathology was determined according to pre-operative diagnosis, intra-operative and pathological assessment. All patients with symptomatic mitral valve disease following an episode of confirmed endocarditis prior to surgery were considered as mitral valve disease secondary to endocarditis.

Concomitant procedures included coronary artery bypass grafting (CABG) in 37 patients, of which 5(11.6%) patients underwent mitral valve repair and 32 (11.7%) patients underwent mitral valve replacement (p = NS). Thirty-seven patients had tricuspid valve repair, including 2(4.7%) with MVrep and 35(12.8%)patients with DVR (p = NS).

Previous cardiac surgery was performed in 144(52.7%) patients in the DVR group and 9(20.9%) patients in the AVR+MVrep group (p = 0.0001), which suggests a greater likelihood to replace the valve at the time of redo operation (Table 2).

**Table 2:** Reoperation patient characteristics

	**DVR**	**AVR+Mvrep**
**n**	144(52.7%)	9(20.9%)
**Age **(median ± SD)	59.2 ± 9.3	56.0 ± 1.6
		
**Previous operation**		
Aortic valve	12(4.4%)	2(4.7%)
Mitral valve	68(24.9%)	6(14%)
Aortic and Mitral valves	57(20.9%)	1(2.3%)
Other than valve	4(1.5%)	0
Unknown	3(1.1%)	0

### Surgical procedure

A total of 589 prosthetic valves were implanted, 316 of these were aortic and 273 mitral. The median implanted valve size in the aortic position was 23 mm(19–29) and in the mitral position 31 mm(25–33).

The operative technique varied depending on the surgeon performing the procedure, but all surgeons used moderate hypothermia (28°C to 32°C) along with cold crystalloid or blood cardioplegia. Mitral valve repair or replacement was the decision of the individual surgeon. Mitral valve repair was classified as all procedures performed in keeping with the techniques as originally described by carpentier [2].

All patients were anticoagulated with warfarin. The goal for the therapeutic range of international normalised ratio (INR) was 2.0 to 3.0 and more recently (1992 onwards), 2.0 to 2.5 for AVR +MVrep and 2.5 to 3.0 for DVR. The changes in INR resulted from data previously published from our unit showing a decrease in haemorrhagic events with no resultant changes in thromboembolic complications for isolated mechanical prosthesis in the aortic position [1]. Until a therapeutic international normalized ratio was achieved, patients were maintained in hospital on either subcutaneous heparin or enoxaparin.

### Data collection and follow up

Follow-up for surviving patients was carried out by telephone interview with the patient, their family physician, or a close relative during a closing interval of 220 days and ranged from 3 months to 21 years (9.03 + 4.82 years). During the follow up period 96 patients died. Overall, 50% of surviving patients were followed to 8.02 years, 36.7% to 10 years and 9.6% to 15 years.

Data for deceased patients were obtained from hospital and family physicians' records as well as from death certificates obtained from the Statistics and Research Agency of Northern Ireland. We adhered to the guidelines of the Society of Thoracic Surgeons [3] for reporting morbidity and mortality after cardiac valvular operations. Follow up of 100% was achieved with a total of 2374 patient years.

### Statistical analysis

Continuous variables were expressed as median + standard deviation. For univariate comparisons the Χ^2 ^test and Fisher's exact probability test were used. A p value of less than 0.05 was considered to be significant. The Kaplan-Meier method was used for actuarial analysis, applying either Breslow's or log-rank significance tests. All operative deaths were included in actuarial analysis. The Statsdirect software package (Statsdirect.com) was used with a compatible desktop computer.

## Results

### In hospital mortality

Seventeen patients (5.4%) died either in hospital or in the first 30 days following operation (range 0 to 40 days). Of these patients 16 had undergone DVR and 1 AVR+MVrep (p = NS). The major causes of hospital death were post-operative low cardiac output (8) or multiple organ failure (2). One death occurred due to fracture of the aortic prosthetic valve. A further death occurred due to rupture of the left ventricle along the posterior atrio-ventricular junction in a patient undergoing DVR following two previous mitral valve procedures.

### Late mortality

Ninety-six patients died during the follow-up period. The causes of death are listed in table 3.

**Table 3:** Late causes of death

**Cause of death**	**DVR(n =)**	**AVR+MVrep(n =)**	**p**
**Valve Related Mortality**	33	5	NS
Failed Heart Transplant	0	1	
**Total Cardiac Deaths**	54	9	NS
			
**Total non-Cardiac Deaths**	28	5	NS
Pneumonia	10	2	
Metastatic Disease	11	2	
Renal Failure	2	0	
Pulmonary Embolism	2	0	
Septicaemia	2	0	
Alcoholic Cirrhosis	1	0	
			
**Total Deaths**	**82**	**14**	NS

### Valve related deaths

We documented a total of thirty-eight deaths as valve-related. Thirty-three occurred in the DVR group: myocardial infarct (9), prosthetic valve endocarditis (8), intracranial haemorrhage (7), thromboembolic CVA (5), mesenteric infarct (2), mitral valve thrombosis (1), and periprosthetic leak (1). There were five valve related deaths within the AVR+MVrep group: myocardial infarct (1), prosthetic valve endocarditis (2), thromboembolic CVA (1), and sudden death (1). The patient who died of sudden death died at home nine years following surgery and no post-mortem was performed. The death was presumed to be valve-related, for the purposes of analysis (p = NS).

### Anticoagulation related complications

Thromboembolic complications were observed in 14 of 273 (5.1%) patients in the DVR group these included: thromboembolic CVA (10), mesenteric infarction (2), and valve thrombosis (2). Of these 14 patients there were 8 deaths (as detailed above). One patient with mitral valve thrombosis had an international normalized ratio of 1.4 times normal on admission and was treated successfully with anticoagulation. There were two episodes of thromboembolic CVA (4.7%) in the AVR+MVrep group with one mortality (p = NS).

Haemorrhagic complications were observed in 12 (4.4%) patients in the DVR group: intracranial haemorrhage (7), gastrointestinal bleed (4), and nose bleed (1). All of the patients with intracranial haemorrhage died, and the other patients all required admission to hospital and blood transfusion. Haemorrhagic complications were observed in 3 of 43 (7.0%) patients in the AVR+Mvrep group: gastrointestinal bleed (1), per vaginal bleed (1), nose bleed(1). There were no mortalities but all required blood transfusion (p = NS)

### Prosthetic valve endocarditis

Twelve patients (12/316) patients were identified as having prosthetic valve endocarditis (PVE). Three patients (3/43;7.0%) in the AVR+MVrep group had PVE, two died and one survives. He remains well after conservative management. In the DVR group there were 9 patients (9/273;3.3%) who developed PVE (p = NS). Eight patients died and one survives following conservative management.

### Structural dysfunction

One patient died on the day of surgery in the DVR group and post mortem showed a fracture of one of the aortic valve leaflets.

### Non-structural dysfunction

One patient in the DVR group required re-operation and further mitral valve replacement six years later for mitral valve leaflet entrapment due to pannus formation.

### Periprosthetic leak

Six patients in the DVR group required re-operation for periprosthetic leak, whilst no patients in the AVR+MVrep group developed a leak (P = NS). Of those patients in the DVR group requiring re-operation, one was for aortic valve periprosthetic leak and five for mitral valve periprosthetic leak.

### Actuarial analysis

At follow up, 203 patients were alive to give a total of 2374 patient years for this study. Actuarial survival, regardless of cause of death, for DVR at 5, 10, 15 years was 79%(CI 74–84), 62%(CI 55–68), 44(CI 34–53) respectively, and 86%(CI75–96), 76%(CI62–90), 57%(CI36–79) respectively for the AVR+MVrep group (p = NS).

Actuarial survival for the patients(n = 163) undergoing either DVR or AVR+MVrep, as a first time cardiac procedure, at 5, 10, 15 years was 81%(CI 74–88), 58%(CI 47–69), 39%(CI 21–56), and 82%(CI69–95), 77%(CI62–93), 58%(CI32–84) for the DVR and AVR+MVrep groups respectively (p = NS).

Cox regression analysis was performed using all patients with the variables; procedure performed, sex, mitral valve pathology, aortic valve pathology and redo procedure. Using a forward selection process, only the variable age was significant (p < 0.001), with all the other variables exceeding p = 0.05. However, these results should be treated with caution given the relatively small sample size.

Age significantly affects survival with a hazard ratio of 1.93 (CI 1.47–2.45) per decade.

## Discussion

Our experience in this 21-year study shows that both DVR and AVR+MVrep lead to excellent long-term survival and low morbidity as comparable to other studies [4-7].

The higher early mortality rate appears less favourable than other studies [8-12]. This may be related to the high proportion of patients in NYHA III-IV. Moreover, there was a high incidence of patients undergoing redo operations (153/316; 48.4%). Although redo operation following previous mitral valve repair is associated with low mortality [13] a number of these patients had previous valve replacement which is associated with a higher operative mortality. Patients undergoing concomitant coronary artery bypass were also included and again this is associated with higher mortality [13]. These data were also analysed for the twenty-one years as a whole. Interestingly, the data for the United Kingdom heart valve registry [14] over the period 1987–1997 estimates early mortality at 9.3% for double valve procedures inclusive of re-operations.

Much of the data recently published comparing DVR to AVR+MVrep considers a heterogeneous population of patients undergoing replacement with various mechanical and bioprosthetic valves [4-6]. A major strength of this study, is that it is confined to a single prosthetic valve and hence removes the confounding influences of the different mechanical valves and the earlier structural deterioration associated with bioprosthetic valves.

Gillinov et al [5] had a population of 813 patients undergoing combined aortic valve replacement with mitral valve procedure. They included all patients undergoing surgery irrespective of the valvular prosthesis used during the 23 year period. In total they identified 301 patients receiving a mechanical aortic prosthesis with associated mitral procedure, compared to 316 in the current study.

Long term survival was comparable in both studies. However, the high incidence of bioprosthetic valve insertion in their study population resulted in a higher re-operation rate, as would be expected. Hamamoto et al [4] also studied a similar population, again regardless of the type of prosthesis used. Additionally, they excluded patients undergoing concomitant coronary artery bypass grafting or other procedure. In comparison, only 135 patients within the DVR (135/299) group received a mechanical prosthesis and 31 patients in the AVR+MVrep group received a mechanical prosthesis in the aortic position.

Remadi et al [6] studied 254 consecutive patients undergoing mechanical valve replacement simultaneously of the aortic and mitral valves. Although all mitral valve replacements were with a SJM prosthesis, only 127 patients received such a valve in the aortic position. They exhibited a higher early mortality rate of 7.08% but noted that following this, long-term outcome for DVR was similar to that for isolated MVR. They too, discovered a high incidence of periprosthetic leak for patients undergoing mechanical DVR.

Freedom from thromboembolic complications at 10 years was similar for each group (DVR 29% (CI 15–43); AVR+MVrep 41% (CI 4–79)). There was also no significant difference in the freedom from haemorrhagic complications resulting from anticoagulation therapy.

This study population exhibited a high number of redo operations following previous cardiac surgery (DVR n = 144; AVR+MVrep = 9). In our experience all patients requiring a redo operation had a previous valve other than a SJM, usually a bioprosthesis, inserted at the time of the first cardiac procedure. Survival analysis of patients undergoing first time cardiac surgery of either DVR or AVR+MVrep was similar at 15 years and was independent of concomitant tricuspid valve repair or coronary artery bypass grafting.

There was one re-operation within the AVR+MVrep group with the patient undergoing a further AVR+MVrep. In this case the aortic valve was initially replaced with a bioprosthetic valve. In contrast, there were 6 re-operations for periprosthetic leak within the DVR group. There were no further valve procedures within the DVR group.

In their study Hamamoto et al [4] found that 49% of patients (39/80) required re-operation following AVR+MVrep. However 34 (87.2%) of these patients required re-operation for deterioration of a bioprosthetic valve in the aortic position or pannus associated with a mechanical AVR.

There was a higher incidence of rheumatic fever in the DVR group suggesting that surgeons were more likely to perform a mitral valve replacement for a stenotic rheumatic mitral valve.

The operative mortality rates were 5.9% and 2.3% respectively for DVR and AVR+Mvrep, suggesting a clinical difference.

As is common to retrospective reviews this study has several limitations. Obviously patients were not randomised to the two treatment modalities. The decision to perform either DVR or AVR+MVrep was dependent on the operating surgeon and thus indications naturally varied according to surgical experience. The absolute numbers of patients were relatively small, but these are at least comparable to recently reported series [4-6].

## Conclusion

**Figure 1:**
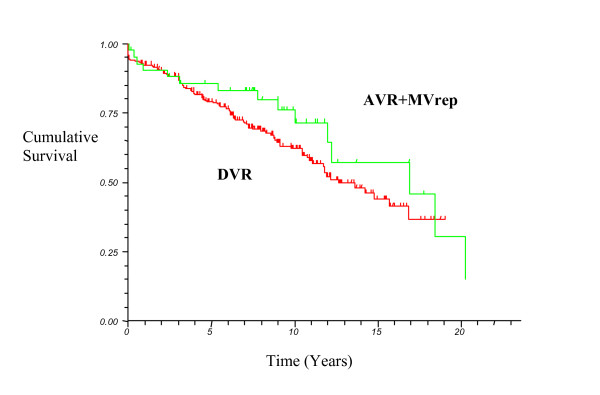
Kaplan-Meier survival curve for all patients undergoing DVR or AVR+MVrep (log rank p = 0.18).

Our previous data shows that survival following DVR is similar to that for isolated MVR - see Fig 1. This current study suggests that patients undergoing combined aortic and mitral valve procedures may benefit from mitral valve repair but unfortunately the available data but did not reach a statistically significant difference in outcome for survival. The authors accept that as a result of the disparity in patient numbers between the two study groups, there is a suggested clinical difference. Both mitral valve repair and replacement are good options for the rheumatic valve patient and the choice should be determined by surgical judgement and experience. Whether repairing or replacing a rheumatic mitral valve at the time of aortic valve replacement makes no significant difference to long-term outcome. These results must be interpreted with caution, however, in the modern era with more myxomatous mitral valve pathology amenable to repair. This study also demonstrates that the use of a mechanical prosthesis in this subgroup of patients results in good long-term survival without the need for reoperation associated with a bioprosthetic valve.

## Competing interests

The author(s) declare that they have no competing interests.

## Authors' contributions

NCMG participated in the design of the study, performed the statistical analysis and coordinated and helped to draft the paper. JMJ and PS participated in the design and helped to draft the paper. SWMG conceived of the study, participated in its design and helped to draft the paper.

## References

[B1] Ibrahim M, O'Kane H, Cleland J, Gladstone D, Sarsam M, Patterson C (1994). The St. Jude medical prosthesis: A 13 year experience. J Thorac Cardiovas Surg.

[B2] Carpentier A (1983). Cardiac valve surgery-the "French correction.". J Thorac Cardiovasc Surg.

[B3] Edmunds LH, Clark RE, Cohn LH, Grunkemeier GL, Miller DG, Weisel RD (1996). Guidelines for reporting morbidity and mortality after cardiac valvular operations. Ann Thorac Surg.

[B4] Hamamoto M, Bando K, Kobayashi J, Tosihiko S, Sasako Y, Niwaya K, Tagusari O, Yagihara T, Kitamura S (2003). Durability and outcome of aortic valve replacement with mitral valve repair versus double valve replacement. Ann Thorac Surg.

[B5] Gillinov MA, Blackstone EH, Cosgrove DM, White J, Kerr P, Marullo A, McCarthy P, Lytle B (2003). Mitral valve repair with aortic replacement is superior to double valve replacement. J Thorac Cardiovasc Surg.

[B6] Remadi JP, Baron O, Tribouilly C, Roussel JC, Al_Habasch 0, Despins P, Michaud JL, Duveau D (2003). Bivalvular mechanical mitral-aortic valve replacement in 254 patients: Long-term results – A 22-year follow-up. Ann Thorac Surg.

[B7] Fiore AC, Swartz MT, Sharp TG, Kesler KA, Barner HB, Naunheim KS, Grunkemeier GL, Moroney DA, Kaiser GC (1995). Double-valve replacement with Medtronic-Hall or St. Jude valve. Ann Thorac Surg.

[B8] Milano A, Guglielmi C, De Carlo M, Di Gregorio O, Borzoni G, Verunelli F, Bortolotti U (1998). Valve-related complications in elderly patients with biological and mechanical aortic valves. Ann Thorac Surg.

[B9] Birkmeyer NJO, Marrin CAS, Morton JR, Leavitt BJ, Lahey SJ, Charlesworth DC, Hernandez F, Olmstead EM, O'Connor GT (2000). Decreasing mortality for aortic and mitral valve surgery in Northern New England. Ann Thorac Surg.

[B10] Armenti F, Stephenson LW, Edmunds H (1987). Simultaneous implantation of St. Jude Medical aortic and mitral prostheses. J Thorac Cardiovasc Surg.

[B11] Remadi JP, Bizouarn P, Baron O, Al Habash O, Despins P, Michaud J, Duveau D (1998). Mitral valve replacement with the St. Jude medical prosthesis: A 15 year follow-up. Ann Thorac Surg.

[B12] Kawachi Y, Matuzaki K, Tominaga R, Yasui H, Tokunaga K (1994). The risks of re-operation for prosthetic valve dysfunction. Surgery Today.

[B13] Jones JM, O'Kane H, Gladstone DJ, Sarsam MA, Campalani G, MacGowan SW, Cleland J, Cran GW (2001). Repeat heart surgery: Risk factors for operative mortality. J Thorac Cardiovasc Surg.

[B14] The United Kingdom heart valve registry report 2001

[B15] Fasol R, Mahdjoobian K, Joubert-Hubner E (2002). Mitral repair in patients with severely calcified annulus: feasibility, surgery and results. J Heart valve Dis.

[B16] Guillinov AM, Cosgrove DM (2002). Mitral valve repair for degenerative disease. J Heart Valve Dis.

